# Pro-Inflammatory diet accounts for higher prevalence of retinopathy in diabetes participants rather than normal glucose and prediabetes: Results from NHANES, 2005–2008

**DOI:** 10.3389/fnut.2022.981302

**Published:** 2023-01-11

**Authors:** Wenjun Pan, Zhuqi Zhang, Yuzhuo Zhang, Haining Lu, Baohua Wang, Shaoyang Zhao, Saimei Li

**Affiliations:** ^1^Postdoctoral Research Station, Guangzhou University of Chinese Medicine, Guangzhou, China; ^2^The Second Affiliated Hospital of Guangzhou University of Chinese Medicine, Guangzhou, China; ^3^Department of Endocrinology, The First Affiliated Hospital of Guangzhou University of Chinese Medicine, Guangzhou, China

**Keywords:** diabetes, retinopathy, dietary inflammatory index, diabetic diets, inflammation

## Abstract

Retinopathy is a chronic inflammatory disease whose prognosis could be improved with dietary interventions. However, the association between a pro-inflammatory diet and the prevalence of retinopathy has not been fully elucidated. We assess the association between the dietary inflammatory index (DII), which is a comprehensive index determining inflammatory potential derived from food parameters according to literature, and the prevalence of retinopathy based on the data from the National Health and Nutritional Examination Survey (NHANES) 2005–2008 involving 2,403 participants. Energy-adjusted DII (E-DII) was not related to the occurrence of retinopathy in the general, non-diabetic, or middle-aged participants. In the diabetic and aged participants, one unit increment of E-DII accounted for 14 and 15% higher the prevalence of retinopathy respectively. The highest E-DII group had a 78 and 79% higher prevalence of retinopathy than the lowest group respectively. After adjusting for several covariables, the highest E-DII group was still associated with a 68% increase in retinopathy in diabetic patients. These results suggest that E-DII is positively associated with the prevalence of diabetic retinopathy among diabetic patients.

## Introduction

There is a growing number of people at risk of retinopathy, whose incidence is estimated to be 34.6% in diabetes, which is a major epidemic worldwide (1). The global prevalence of age-related macular degeneration is 8.69% mapped to an age range of 45–85 years (2). Retinopathy causes progressive vision loss, which not only has widespread effect on the quality of life but also poses pressure on the social economy.

Retinopathy is a chronic inflammatory disease manifested by increased vascular permeability, edema, infiltration of inflammatory cells, neovascularization, and expression of angiogenic factors (3). In addition, intravitreal anti-VEGF agent injection has shown promising therapeutic effects, raising the anti-inflammation strategy for retinopathy (4). Recently, it is reported that diet and lifestyle interventions could improve the prognosis of retinopathy (5).

The dietary inflammatory index (DII) is a literature-derived, population-based index to determine the inflammatory potential of diets (6). Forty-five food parameters were identified to be related to six inflammatory biomarkers: IL-1β, IL-4, IL-6, IL-10, TNF-α, and C-reactive protein, based on the dietary algorithm developed from nearly 6,500 articles (6). Although several studies have established the association between DII and Diabetes Mellitus (DM) as well as its complications (7–10), there is no report regarding the relationship between DII and retinopathy. Herein, we assess the association between DII and retinopathy especially DR using the National Health and Nutrition Examination Survey (NHANES) database. Moreover, we did subgroup analysis and interaction tests regarding age, gender, diabetic status, hypertension, and other demographic and metabolic factors, to identify the specific population among whom DII has a relatively independent association with the prevalence of retinopathy.

## Methods

### Study population

NHANES is a nationwide and ongoing cross-sectional survey managed by the National Center for Health Statistics at the US Centers for Disease Control and Prevention. It conducts a repeated 2-year cycle test with a complex multistage probability sampling design, and all sample weights are designed to represent data for the civilian non-institutionalized US population. The NHANES research protocol was approved by the institutional review board and included the written, informed consent of all participants, following the principles of the Declaration of Helsinki. Data in this study were all obtained from NHANES, publicly available at https://www.cdc.gov/nchs/nhanes/ (accessed date: 27 March 2022).

**Figure 1:**
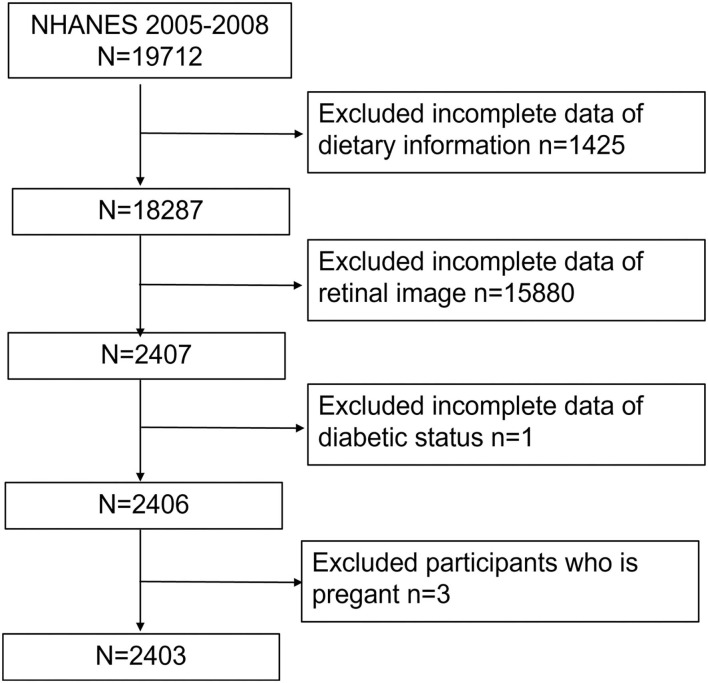
Flowchart of sample selection from NHANES 2005–2008.

Our study was based on a 4-year NHANES survey cycle, 2005–2008, since this cycle included full information on retinopathy based on a retinal imaging exam. After excluding participants without necessary dietary conditions (*n* = 1,425), retinal image results (*n* = 15,880), or diabetic status (from questionnaire and plasma glucose and HbA1c test, *n* = 1) as well as pregnant women (*n* = 3), we finally included 2,403 participants (Figure 1).

### Definition of participants, exposure, and outcomes

For the present study, subjects who declared to have a previous DM physician-diagnosis or who have fasting glucose ≥126 mg/dL, HbA1c (%) ≥6.5, or OGTT ≥200 mg/dL, were defined as DM. Participants who declared to have a pre-DM physician diagnosis or who have fasting glucose ≥100 mg/dL, HbA1c (%) ≥5.7, or OGTT ≥140 mg/dL were defined as pre-diabetic. Furthermore, subjects who reported no DM or pre-diabetes history with normal glucose and HbA1c test were considered normal. The whole population was constituted of 1,089 normal glucose, 823 prediabetes, and 491 DM participants.

DII was designed as the exposure variable. Dietary intake was documented and validated utilizing the 24-h dietary history interview at the Mobile Examination Center. The dietary data were obtained at the mobile examination center and were validated by the Nutrition Methodology Working Group. The 24-h dietary recall data were used to calculate the DII score according to the calculating protocol published by Shivappa et al. (6). In our study, 27 of the 45 food parameters were available to calculate DII in the 2005–2008 NHANES cycle, including alcohol, protein, fiber, β-carotene, cholesterol, carbohydrates, energy, fats, n-3 fatty acid, n-6 fatty acid, poly-unsaturated fatty acid, mono-unsaturated fatty acid, saturated fat, thiamin, magnesium, zinc, selenium, iron, riboflavin, folic acid, vitamin A, vitamin B-6, vitamin B-12, vitamin C, vitamin D, vitamin E, caffeine, and niacin. DII calculation formula is shown below. Each value of the 27 parameters above was subtracted from the global daily mean intake and divided by its standard deviation, both of which were available in N. Shivappa's paper (6). Then the value was converted to a percentile score, multiplied by 2, and subtracted by 1 to achieve symmetrical distribution. The percentile value of each component was multiplied by the corresponding overall inflammatory effect score, which was summed up to get the overall DII score for each participant. The higher positive DII score indicated a more pro-inflammatory diet, while a lower negative DII score suggested an anti-inflammatory effect of diet. To control for the effect of total energy intake, we used the energy-adjusted dietary inflammatory index (E-DII) wherein we calculated all the food parameters per 1,000 kcal of consumption. In this study, the E-DII score was served as a continuous variable and then categorized into tertiles from the total sample for further analysis.

Calculation formula of DII:

Z score = (24 h intake of each component—global daily intake) / standard deviation of global daily intake

Z score^1^ = Z score → converted to a percentile score ×2 −1

DII = *Σ* Z score^1^ × overall inflammatory effect score of each component

According to the severity scale provided by the Early Treatment for Diabetic Retinopathy Study (ETDRS), retinopathy is characterized by hard exudates, vitreous hemorrhage, cotton-wool spots, and intra-retinal microvascular changes over retinal tissue (11). A non-mydriatic fundus photography (TRC-NW6S; Topcon, Tokyo, Japan) was used for detecting DR in the survey based on the NHANES Digital Grading Protocol. In NHANES, participants aged 40 years and older were eligible for retinal imaging. The detailed information can be found in the NHANES Ophthalmology Procedures Manual available on the NHANES website.

### Covariates assessment

We incorporated age, body mass index (BMI, kg/m^2^), race ethnicity, educational level, smoking status, family poverty-to-income ratio (PIR), marital status, hypertension, and high cholesterol as covariates which were available in NHANES questionnaire data or demographic data. Participants aged 65 years and older were considered as aged. Participants with BMI equal to or higher than 25 were overweight according to the World Health Organization standards. The smoking status was classified as never, former smoker, and current smoker based on the “Smoking—Cigarette Use” section in the NHANES Questionnaire data. PIR < 1.00 means household income below the poverty threshold, while PIR higher than 3.00 means household income more than triple the poverty threshold. Participants who were divorced, widow, or lived separately were considered to be living separately. Hypertension and high cholesterol were considered if the participants were ever told they had high blood pressure or cholesterol level.

### Statistical analysis

The descriptive analysis was summarized according to the subjects' retinal status. Continuous variables were presented as mean ± SD and analyzed by Student's *t*-tests. Categorial variables were presented as percentages and analyzed by Chi-square tests. The relationship between E-DII and retinopathy was examined with a logistic regression model. Subgroup analysis was also conducted by categories including gender, age, race ethnicity, educational level, marital status, family income, smoking status, BMI, diabetic status, hypertension, and high cholesterol. Interaction terms, such as demographic parameters, diabetic status, and so on, were added to test the heterogeneity. Then we categorized the participants into 3 groups by the tertiles of E-DII and did logistic regression analysis for the association as well as the Wald test to assess P for trend. A multivariate linear regression was used for the relationship between E-DII and retinopathy after being stepwise adjusted for the potential confounding factors. Lastly, weighted generalized additive models and smooth curve fittings were used to address the non-linear association between E-DII and DR in DM patients. All analyses were performed with the software package R (http://www.R-project.org, The R Foundation, access on 26 April 2022) and EmpowerStats (www.empowerstats.com access on 26 April 2022).

## Results

### Baseline characteristics of participants

Table 1 lists characteristics information of 2,403 eligible participants with (*n* = 305) and without retinopathy (*n* = 2,098). Participants with retinopathy tended to be older, low-educated, and overweight with no high income. The prevalence of DM, hypertension, or dyslipidemia was higher in people with retinopathy than in those without. Non-Hispanic Whites had a lower prevalence of retinopathy than other race ethnicities. E-DII was significantly higher in participants with retinopathy than in those without. There was no obvious difference between retinopathy and non-retinopathy in marital and smoking status. In Supplementary Table S1, we found that there were significant differences in gender, race ethnicity, educational level, marital status, family PIR, diabetic status, hypertension, and smoking status among participants with different levels of E-DII.

**Table 1:** Baseline characteristics of participants in the 2005–2008 NHANES.

**Characteristics**		**Retinopathy**		***P*-value**
		**No (*n* = 2098)**	**Yes (*n* = 305)**	
DII		0.60 ± 1.93	0.79 ± 1.81	0.145
E-DII		0.77 ± 3.68	0.87 ± 1.68	0.048
Male		1061 (50.57%)	174 (57.05%)	0.034
Age	< 65	1427 (68.02%)	182 (59.67%)	0.004
	≥65	671 (31.98%)	123 (40.33%)	
Race ethnicity	Non-Hispanic White	1235 (58.87%)	127 (41.64%)	< 0.001
	Other	863 (41.13%)	178 (58.36%)	
Educational level	< High school	549 (26.17%)	116 (38.03%)	< 0.001
	High school or equivalent	520 (24.79%)	76 (24.92%)	
	> High school	1028 (49.00%)	113 (37.05%)	
	Missing	1 (0.05%)	0 (0.00%)	
Marital status	Married	1342 (63.97%)	203 (66.56%)	0.548
	Live separated	610 (29.08%)	87 (28.52%)	
	Never married	144 (6.86%)	15 (4.92%)	
	Missing	2 (0.10%)	0 (0.00%)	
Family PIR	< 1	266 (12.68%)	48 (15.74%)	0.004
	1 to 3	790 (37.65%)	138 (45.25%)	
	>3	949 (45.23%)	104 (34.10%)	
	Missing	93 (4.43%)	15 (4.92%)	
Diabetic status	Normal	1007 (48.00%)	82 (26.89%)	<0.001
	Prediabetes	754 (35.94%)	69 (22.62%)	
	Diabetes	337 (16.06%)	154 (50.49%)	
Hypertension	Yes	888 (42.33%)	181 (59.34%)	<0.001
	No	1206 (57.48%)	123 (40.33%)	
	Missing	4 (0.19%)	1 (0.33%)	
High cholesterol	Yes	820 (47.29%)	144 (56.69%)	0.012
	No	904 (52.13%)	110 (43.31%)	
	Missing	10 (0.58%)	0 (0.00%)	
Smoking	Never	949 (45.23%)	143 (46.89%)	0.449
	Ever	702 (33.46%)	109 (35.74%)	
	Current	446 (21.26%)	53 (17.38%)	
	Missing	1 (0.05%)	0 (0.00%)	
BMI	< 25	575 (27.41%)	57 (18.69%)	0.005
	≥25	1507 (71.83%)	246 (80.66%)	
	Missing	16 (0.76%)	2 (0.66%)	

For continuous variables, P-value was calculated by weighted t-test. For categorical variables, P-value was calculated by weighted chi-square test. DII, dietary inflammatory index; E-DII, energy-adjusted dietary inflammatory index; PIR, poverty -to- income ratio; BMI, Body Mass Index.

### Association between E-DII score and retinopathy

As shown in Table 2, in the whole population, E-DII was not significantly associated with the prevalence of retinopathy when it was taken as a continuous variable (OR = 1, 95%CI: 0.97–1.04). Then, we did subgroup analysis according to gender, age, race ethnicity, educational level, marital status, family income, diabetic status, hypertension, dyslipidemia, smoking status, and BMI. E-DII was significantly associated with the prevalence of retinopathy in DM and aged (age ≥ 65) participants. One unit increase in E-DII significantly accounted for 14 and 15% increments in the prevalence of retinopathy in DM patients (OR = 1.14, 95%CI: 1.02–1.26) and aged participants (OR = 1.15, 95%CI: 1.03, 1.28), respectively. The log likelihood ratio test proved that there was a significant interaction between E-DII and retinopathy among different diabetic status and age groups (both P for interaction <0.05), while we found no significant difference among other groups (all P for interaction > 0.05).

**Table 2:** The association between E-DII and retinopathy by different subgroups.

**Population**	***N***	**Retinopathy (OR,95%CI)**	**P for interaction**
**Total**	2403	1.00 (0.97, 1.04)	
**Gender**			0.9795
Male	1235	1.01 (0.89, 1.14)	
Female	1168	1.01 (0.98, 1.04)	
**Age**			0.0146^*^
< 65	1609	0.99 (0.94, 1.05)	
≥65	794	1.15 (1.03, 1.28)^*^	
**Race ethnicity**			0.359
Non-Hispanic White	1362	1.00 (0.95, 1.05)	
Other	1041	1.04 (0.96, 1.13)	
**Educational level**			0.2153
< High school	665	1.05 (0.96, 1.16)	
High school or equivalent	596	0.95 (0.80, 1.11)	
>High school	1141	1.07 (0.95, 1.21)	
**Marital status**			0.6404
Married	1545	1.00 (0.97, 1.03)	
Live separated	697	1.06 (0.95, 1.18)	
Never married	159	0.94 (0.68, 1.30)	
**Family PIR**			0.3969
< 1	314	1.03 (0.90, 1.18)	
1 to 3	928	0.99 (0.94, 1.05)	
>3	1053	1.06 (0.92, 1.22)	
Missing	108	1.27 (0.92, 1.76)	
**Diabetic status**			0.0345^*^
Normal	1089	0.96 (0.82, 1.12)	
Prediabetes	823	0.92 (0.78, 1.09)	
Diabetes	491	1.14 (1.02, 1.26)^*^	
**Hypertension**			0.4829
Yes	1069	1.00 (0.98, 1.03)	
No	1329	0.96 (0.85, 1.09)	
**High cholesterol**			0.1134
Yes	964	1.09 (0.99, 1.21)	
No	1014	1.00 (0.96, 1.04)	
**Smoking**			0.2939
Never	1092	1.07 (0.98, 1.17)	
Ever	811	1.00 (0.95, 1.04)	
Current	499	1.07 (0.91, 1.27)	
**BMI**			0.3021
< 25	632	1.09 (0.94, 1.26)	
≥25	1753	1.00 (0.97, 1.03)	
Missing	18	1.12 (0.65, 1.94)	

E-DII was considered as a continuous variable. Univariate analysis was conducted to assess the association between E-DII and the prevalence of retinopathy in the general population and each subgroup. Log likelihood ratio test was performed to identify the interaction effect of different subgroups.

* P < 0.05. PIR, poverty -to- income ratio; BMI, Body Mass Index.

Then we categorized aged and DM participants into three groups by tertiles of E-DII. The highest E-DII group had a 78 and 79% higher prevalence of DR as compared to the lowest group (OR = 1.78, 95%CI:1.11–2.85 and OR = 1.79, 95%CI:1.11–2.88) in DM patients and aged participants, respectively (Table 3). Furthermore, the occurrence of retinopathy had a significant increasing trend across the tertiles of E-DII for both DM and aged participants (both P for trend < 0.05).

**Table 3:** Association between E-DII and retinopathy in the aged and diabetes participants according to different models.

**Population**	**E-DII**	**Unadjusted**	**Model I**	**Model II**	**Model III**
	Continuous	1.15 (1.03, 1.28)^*^	1.13 (1.01, 1.26)^*^	1.09 (0.96, 1.22)	1.10 (0.96, 1.26)
	Tertile 1	1	1	1	1
Age≥65	Tertile 2	1.41 (0.85, 2.35)	1.43 (0.87, 2.36)	1.35 (0.81, 2.25)	1.31 (0.74, 2.32)
	Tertile 3	1.79 (1.11, 2.88)^*^	1.60 (0.97, 2.63)	1.39 (0.83, 2.33)	1.56 (0.88, 2.77)
	P for trend	0.036^*^	0.079	0.252	0.135
	Continuous	1.14 (1.02, 1.26)^*^	1.12 (1.00, 1.26) ^*^	1.13 (1.00, 1.27)^*^	1.12 (0.98, 1.27)
	Tertile 1	1	1	1	1
Diabetes	Tertile 2	1.27 (0.76, 2.13)	1.14 (0.70, 1.86)	1.19 (0.72, 1.97)	1.26 (0.72, 2.20)
	Tertile 3	1.78 (1.11, 2.85)^*^	1.63 (1.00, 2.65)^*^	1.68 (1.02, 2.77)^*^	1.70 (0.98, 2.94)
	P for trend	0.022^*^	0.043^*^	0.037^*^	0.057

E-DII was considered as a categorial variable. Multiple regression analysis was conducted to assess the association between E-DII and the prevalence of retinopathy in the aged and diabetes subgroups in different statistical models.

For aged, Model I was adjusted for gender and race ethnicity; Model II were adjusted for gender, race ethnicity, educational level, marital status, family PIR, smoking status, and BMI. Model III was adjusted for gender, race ethnicity, educational level, marital status, family PIR, smoking status, BMI, hypertension, diabetic status, and high cholesterol.

For diabetes, Model I was adjusted for gender, age, and race ethnicity; Model II was adjusted for gender, age, race ethnicity, educational level, marital status, family PIR, smoking status, and BMI. Model III were adjusted for gender, age, race ethnicity, educational level, marital status, family PIR, smoking status, BMI, hypertension, and high cholesterol.

* P < 0.05. E-DII, energy-adjusted dietary inflammatory index.

After adjusted for age, gender, and race ethnicity, 1 unit increment in the E-DII score accounted for a 12% (OR = 1.12 95%CI: 1–1.26) increment in the prevalence of DR in DM people. The prevalence of DR in the highest tertile is 63% higher than in the lowest tertile group (OR=1.63 95%CI: 1–2.65). After further adjusted for educational level, marital status, family income, smoking status, and BMI, the statistical significance remains with 1 unit increment in the DII score accounting for a 13% (OR = 1.13 95% CI, 1.00–1.27) increase in the prevalence of DR. The prevalence of DR in the highest tertile is 68% higher than in the lowest tertile (OR = 1.68 95% CI, 1.02–2.77). There is a significantly increased risk for retinopathy across the tertiles of E-DII in all the models above for DM patients (all P for trend < 0.05). However, the association between E-DII and DR lost significance with hypertension and dyslipidemia included in the covariables although E-DII induced a 12% increase in the prevalence of DR with a 1 unit increment (OR = 1.12 95% CI, 0.98–1.27). In aged participants, 1 unit increment in the E-DII score accounted for a 13% (OR = 1.13, 95%CI: 1.01–1.26) increment in the prevalence of retinopathy after adjusted for gender and race ethnicity. However, E-DII was not significantly associated with retinopathy with additional covariables whenever it was taken as a continuous or categorial variable in aged participants.

**Figure 2:**
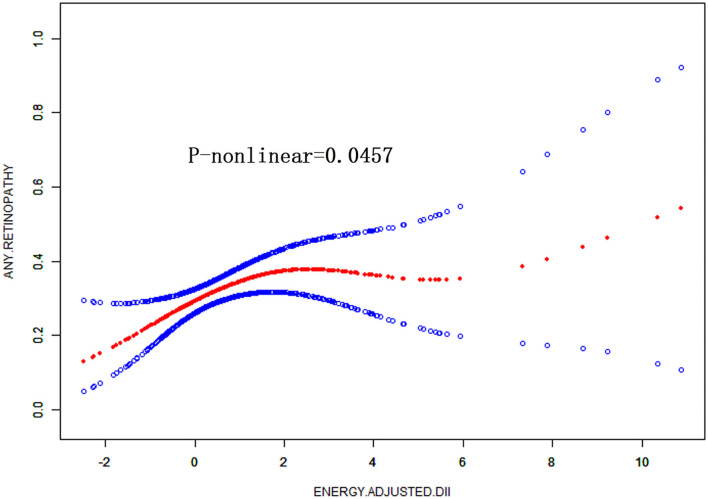
Non-linear relationship between DII and the prevalence of retinopathy in diabetes by the generalized additive model after adjusted for gender, age, race ethnicity, educational level, marital status, family PIR, smoking status, and BMI.

We conducted a log-likelihood ratio test comparing the one-line linear regression model with a two-piecewise linear model in DM patients, which indicated a non-linear relationship between DII and retinopathy (*P* < 0.01) (Figure 2, Table 4). When E-DII is < −0.87, there is a significant positive correlation between E-DII and retinopathy. When E-DII is higher than −0.87, the curve tends to be flat, and E-DII does not give rise to a higher prevalence of DR.

**Table 4:** Threshold effect analysis of E-DII on retinopathy using the two-piecewise linear regression model.

	**OR (95%CI)**	***P*–value**
Fitting by the standard linear model	1.13 (1.00, 1.27)	0.0457
Fitting by the two–piecewise linear model		
Inflection point	−0.87	
E–DII < −0.87	22.94 (1.07, 493.09)	0.0453
E–DII > −0.87	1.07 (0.94, 1.21)	0.3042
Log–likelihood ratio	0.006	

Gender, age, race ethnicity, educational level, marital status, family PIR, smoking status, and BMI were adjusted.

## Discussion

In the study based on NHANES data, we observed no significant association between E-DII and the prevalence of retinopathy in the general population. In the subgroup analysis, one unit increase of E-DII gives rise to a significantly higher prevalence of retinopathy in aged and DM participants, respectively. The most pro-inflammatory diet induced a higher prevalence of retinopathy than the most anti-inflammatory diet group in aged and DM participants, respectively. The relationship remains significant after adjusted for age, gender, race ethnicity, educational level, marital status, PIR, smoking status, and BMI in DM patients, which is a non-linear relationship.

Consistent with previous studies, which showed an anti-inflammatory diet alleviated retinopathy in aged or diabetic participants, we found a diet with anti-inflammatory potential was associated with a decreased prevalence of retinopathy in DM or aged participants. Adherence to a Mediterranean diet or antioxidant supplement with Omega-3 unsaturated fatty acids and vitamins could improve age-related macular degeneration and DR (12–17). DII is an integrated index to evaluate dietary inflammatory potential, which might synthetically reflect consumption lifestyle and give instructions to patients at high risk of retinopathy.

Both DR and age-related macular degeneration are chronic inflammatory diseases that involves the activation of the microglia, monocytes-macrophages, neutrophils, and lymphocytes as well as the secretion of inflammatory mediators, including IL-6, TNF-α, MCP-1, and VEGF (18, 19). All of these cause damage to endothelium, pericytes, and ganglion cells, which promotes vascular permeability and neovascularization (20, 21). DM is closely associated with gut microbiota dysbiosis, which destructs gut mucosa barrier integrity, enhances intestinal permeability, and increases plasma endotoxins (22, 23). Recently, DII was found to be negatively associated with enterolignans, a potential marker for microbiota diversity, which indicated anti-inflammatory diet might protect against DR through the improvement of gut flora and systematic inflammation (24).

DII is crudely associated with retinopathy in aged participants when it is taken as a categorial or continuous variable. After adjusted for gender and race ethnicity, one unit increment of E-DII significantly accounted for 13% increase in the prevalence of retinopathy, but it lost statistical significance after adjusted for other confounding factors in other models. DII is associated with multiple metabolic diseases including obesity, DM, and cardiovascular disease (25). In our study, DII was crudely and positively associated with BMI, hypertension, and high cholesterol, which are risk factors for DR (26, 27). Although after adjusted for demographic factors, smoking status, and BMI, the association between E-DII and DR still exists. The association lost statistical significance with hypertension and high cholesterol included in the covariables. Hence, the association between E-DII and DR cannot rule out the effect of hypertension and high cholesterol. In addition, E-DII is not only associated with the prevalence of DM but also positively related to the severity of DM and insulin resistance (7, 8). Our study provides evidence that E-DII is associated with DR, which is a diabetic complication found in 22.27% of DM patients (28). Furthermore, E-DII was not associated with the presence of retinopathy in normal glucose and prediabetes, which implied that the presence of retinopathy was induced by the synergistic effect of inflammation and hyperglycemia or insulin resistance (7).

To our knowledge, this is the first study exploring the association between DII and retinopathy. However, there are some inevitable limitations. Firstly, out of the 45 parameters, we took the 27 parameters available in NHANES, which might not reflect the whole dietary inflammatory potential. In addition, DII originated from 24-h dietary intake recall, which would give rise to recall bias. We did not include glucose control levels in the covariates, as the ideal glucose control level is not consistent for different age groups. So was medication data, which was hard to extract from the NHANES database. So, prospective cohort studies are needed to verify the causation between E-DII and retinopathy in DM patients independent of these confounding factors. Finally, we did not do survey design analysis in this study because we aimed to assess the association between E-DII and retinopathy rather than epidemiology surveys, which focus on the prevalence of some diseases in the whole population. Due to the inclusion and exclusion criteria that excluded lots of samples, our data might not represent the general population of the United States.

## Conclusion

A pro-inflammatory diet is positively associated with the prevalence of retinopathy in patients with DM rather than people with normal glucose or prediabetes. To prevent retinopathy, it is advisable for patients with DM to take an anti-inflammatory diet.

## Data availability statement

The datasets presented in this study can be found in online repositories. The names of the repository/repositories and accession number(s) can be found below: www.cdc.gov/nchs/nhanes.

## Ethics statement

The studies involving human participants were reviewed and approved by the studies involving human participants were reviewed and approved by the NCHS Ethic Review Board. The patients/participants provided their written informed consent to participate in this study.

## Author contributions

SL directed the study. WP designed the study. WP, ZZ, and YZ did the data extraction and analysis. HL plotted the figures. WP and YZ wrote the article. SZ guided and gave constructive advice during revision. All authors contributed to the article and approved the submitted version.
