# Supplementary material for: Pro-Inflammatory diet accounts for higher prevalence of retinopathy in diabetes participants rather than normal glucose and prediabetes: Results from NHANES, 2005–2008
Source: Front Nutr. 2023 Jan 11;9:981302. doi: 10.3389/fnut.2022.981302 (PMC9875592; doi:10.3389/fnut.2022.981302)
Supplement: Supplementary file 1 [file Table_1.DOCX]

Supplemental table 1. Baseline characteristics of participants according to the tertiles of energy-adjusted dietary inflammatory index in the 2005–2008 NHANES.

| **Characteristics** | **E-DII** | | | ***P*-value** |
| --- | --- | --- | --- | --- |
|  | **T1** (n=801) | **T2** (n=801) | **T3** (n=801) |  |
| **DII** | -1.61 ± 1.05 | 0.87 ± 0.65 | 2.61 ± 0.72 | <0.001 |
| **E-DII** | -0.66 ± 0.46 | 0.43 ± 0.31 | 2.59 ± 5.55 | <0.001 |
| **Retinopathy** |  |  |  | 0.06 |
| No | 713 | 703 | 682 |  |
| Yes | 88 | 98 | 119 |  |
| **Male** | 492 | 467 | 276 | <0.001 |
| **Age** |  |  |  | 0.091 |
| < 65 | 534 | 558 | 517 |  |
| ≥65 | 267 | 243 | 284 |  |
| **Race Ethnicity** |  |  |  | <0.001 |
| Non-Hispanic White | 496 | 451 | 415 |  |
| Other | 305 | 350 | 386 |  |
| **Educational level** |  |  |  | <0.001 |
| < High school | 187 | 214 | 264 |  |
| High school or equivalent | 177 | 189 | 230 |  |
| > High school | 437 | 398 | 306 |  |
| Missing | 0 | 0 | 1 |  |
| **Marital status** |  |  |  | <0.001 |
| Married | 566 | 533 | 446 |  |
| Live separated | 191 | 212 | 294 |  |
| Never married | 43 | 55 | 61 |  |
| Missing | 1 | 1 | 0 |  |
| **Family PIR** |  |  |  | <0.001 |
| < 1 | 88 | 85 | 141 |  |
| 1 to 3 | 266 | 312 | 350 |  |
| > 3 | 413 | 365 | 275 |  |
| Missing | 34 | 39 | 35 |  |
| **Diabetic status** |  |  |  | <0.001 |
| Normal | 403 | 356 | 330 |  |
| Prediabetes | 254 | 297 | 272 |  |
| Diabetes | 144 | 148 | 199 |  |
| **Hypertension** |  |  |  | 0.009 |
| Yes | 322 | 353 | 394 |  |
| No | 477 | 447 | 405 |  |
| Missing | 2 | 1 | 2 |  |
| **High cholesterol** |  |  |  | 0.715 |
| Yes | 315 | 335 | 314 |  |
| No | 351 | 323 | 340 |  |
| Missing | 4 | 3 | 3 |  |
| **Smoking Status** |  |  |  | <0.001 |
| Never | 351 | 369 | 372 |  |
| Ever | 322 | 245 | 244 |  |
| Current | 128 | 187 | 184 |  |
| Missing | 0 | 0 | 1 |  |
| **BMI** |  |  |  | 0.12 |
| <25 | 236 | 199 | 197 |  |
| ≥25 | 561 | 596 | 596 |  |
| Missing | 4 | 6 | 8 |  |

For continuous variables, *P*-value was calculated by weighted t-test. For categorical variables, *P*-value was calculated by weighted chi-square test. Abbreviations: DII, dietary inflammatory index; E-DII, energy-adjusted dietary inflammatory index; PIR, poverty -to- income ratio; and BMI, Body Mass Index.
